# Comparative evaluation of resiliency of mono-poly coated two different acrylic based tissue conditioners - An *in vitro* study

**DOI:** 10.6026/973206300211775

**Published:** 2025-06-30

**Authors:** Apurva Kurhade, Vidushi Jayaswal, Mukesh Sisodiya, Harsh Chansoria, Alka Gupta, Shivani Gupta

**Affiliations:** 1 Department of Prosthodontics and Crown & Bridge, Government College of Dentistry, Indore, Madhya Pradesh, India; 2 Department of Orthodontics and Dentofacial Orthopaedics, Shri Dharmasthala Manjunatheshwara College of Dental Sciences, Dharwad, Karnataka, India

**Keywords:** Acrylic resin, artificial saliva, monopoly coating, resiliency, tissue conditioners

## Abstract

The resiliency of two monomer-coated acrylic-based tissue conditioners over 28 days is compared using an *in vitro* study. Sixty resin specimens were prepared and tested at five intervals using a durometer to measure viscoelastic properties. Initially, Orthoplast Acryton exhibited higher resiliency, but Maarc Soft Liner showed better performance from Day 7 onwards. Both materials demonstrated comparable resiliency after two weeks, suggesting stabilization over time. Mono-poly coating effectively preserved the softness and elasticity, supporting clinical use for short- to medium-term denture conditioning.

## Background:

Tissue conditioners and soft liners are resilient, pliable materials widely employed in prosthodontic practice. They serve multiple purposes, including functional impression recording, temporary relining of ill-fitting dentures and promoting the healing of inflamed or distorted mucosal tissues. Additionally, they are frequently utilized following implant placement during the healing phase [1, 2]. Clinically, tissue conditioners have demonstrated efficacy in improving the fit of dentures, enhancing patient comfort and aiding in tissue recovery [3, 4]. The functional performance of these materials is governed primarily by their viscoelastic behavior, which manifests after gelation. These viscoelastic characteristics must be appropriate and adaptable for their intended clinical application [5]. The inherently moist oral environment significantly impacts the performance and longevity of soft liners. Plasticizers such as ethanol and esters tend to leach out when exposed to saliva or water, while the polymeric matrix absorbs moisture [6]. This interaction alters the surface texture, making it rough and less flexible over time [7]. Consequently, the clinical lifespan of tissue conditioners is often limited to a few weeks, necessitating frequent replacements. To mitigate this limitation and extend the functional duration of soft liners, various surface coating agents-including Palaseal, fluorinated copolymers and Monopoly have been developed. These coatings serve to preserve surface integrity and maintain material resiliency [8]. Therefore, it is of interest to investigate two commercially available soft liners, both coated with Monopoly solution and compares their resiliency over 28 days using a standardized *in vitro* protocol.

## Materials and Methods:

## Preparation of resin specimens:

A total of 60 cuboidal resin specimens, each measuring 10 mm x 10 mm x 2 mm, were fabricated using a heat-cured acrylic resin (Dentsply, Germany). A powder-to-liquid ratio of 2.5:1 was employed and the resin was processed in a water bath at 74°C for eight hours following standard flasking procedures.

## Specimen grouping:

Group A: 30 specimens lined with Orthoplast Acryton Soft Liner (mixed at a 1 g: 1mL powder-to-liquid ratio), then coated with Monopoly solution. These were subdivided into five subgroups (A1-A5) and tested at 24 hours, on Days 7, 14, 21 and 28, respectively.

Group B: 30 specimens lined with the Soft Liner Kit (Maarc Dental), prepared similarly and coated identically with Monopoly. These were also divided into five subgroups (B1-B5) and tested at the same intervals.

## Preparation of monopoly solution:

The monopoly solution was formulated by combining clear methyl methacrylate polymer and a chemically activated methyl methacrylate monomer in a 1:10 powder-to-liquid ratio. The mixture was heated in a 55°C water bath and stirred for 8-10 minutes until a viscous, syrup-like consistency was achieved. The solution was stored at 4°C in an opaque container. Three uniform coats were applied to each specimen using a high-quality brush, with each layer allowed to dry for 4-5 minutes. The coated specimens were subsequently immersed in artificial saliva.

## Assessment of resiliency:

Resiliency was measured using a durometer calibrated according to the ASTM scale (0-100 units), where lower values denote higher resiliency. Measurements were taken for each specimen across the specified intervals.

## Results:

The present study evaluated the resiliency of monopoly-coated tissue conditioners from two different brands (Group A: Orthoplast Acryton and Group B: Maarc Soft Liner) over 28 days using durometer readings. Lower durometer values indicated greater material resiliency. The data were analyzed both between groups (intergroup comparison) and within each group over time (intragroup comparison).

## Intergroup comparison:

As shown in the first table, Group A exhibited significantly higher resiliency on Day 1 (Mean = 41.33) compared to Group B (Mean = 34.58), with a p-value of 0.025, indicating statistical significance. This suggests that Orthoplast Acryton initially had better cushioning ability. However, by Day 7, Group B showed a marked increase in resiliency (Mean = 53.58), significantly higher than Group A (Mean = 32.50), with a highly significant p-value < 0.001. This reversal indicates that Maarcs' soft liner responded better after the first week of immersion in artificial saliva. From Day 14 to Day 28, the resiliency values of both groups became statistically comparable, as reflected by non-significant p-values (p > 0.05) across these intervals. This convergence suggests that both materials tend to stabilise and perform similarly over time (Table 1 - see PDF) (Figure 1 - see PDF).

## Intragroup comparison Group A:

As presented in the second table, Group A demonstrated a significant decrease in resiliency from Day 1 (Mean = 41.33) to Day 7 (Mean = 32.50), with a p-value = 0.002. This indicates a rapid decline in flexibility during the first week. However, no significant differences were found between Day 1 and subsequent time points (Days 14, 21 and 28), suggesting that the material stabilized after the initial drop (Table 2 - see PDF).

## Intragroup comparison Group B:

The third table shows that Group B experienced a significant increase in resiliency from Day 1 (Mean = 34.58) to both Day 7 (Mean = 53.58) and Day 14 (Mean = 52.50), with p-values = 0.001 and 0.002, respectively. These findings indicate a rapid and substantial improvement in material softness and elasticity. Although a slight decrease in resiliency was observed at Day 21 (Mean = 44.92), this change was not statistically significant (p = 0.064). By Day 28, resiliency remained significantly higher than baseline (Mean = 43.92; p = 0.003), indicating a sustained improvement over time (Table 3 - see PDF).

## Discussion:

The present *in vitro* study aimed to evaluate and compare the resiliency of monopoly-coated tissue conditioners from two different commercially available brands over 28 days. Resiliency, a crucial property for soft liners used in denture base relining, plays a significant role in providing cushioning and reducing trauma to the underlying tissues. The ability of a soft liner to retain its viscoelastic properties over time is crucial for its long-term clinical success [9]. In our study, Group A specimens (Orthoplast Acryton) exhibited significantly higher resiliency at Day 1 compared to Group B (Soft Liner Kit by Maarc). This initial finding suggests that Orthoplast Acryton may have superior immediate viscoelastic properties when coated with monomer, potentially providing better early adaptation and cushioning during the immediate post-insertion period. However, this trend was not sustained over time. By Day 7, Group B showed significantly higher resiliency than Group A, indicating a reversal in performance. This suggests that while Group A initially possesses better properties, it may undergo rapid degradation in the first week due to plasticizer loss or water absorption, as previously indicated by Sun *et al.* [10].

Interestingly, from Day 14 onwards, no statistically significant difference in resiliency was noted between the two groups. This indicates a convergence in their viscoelastic behavior over time. The stabilization of values after two weeks may be attributed to the equilibrium being achieved in the exchange of fluids and plasticizers with the surrounding artificial saliva medium, a common occurrence with soft liners exposed to moist environments [11]. Within-group comparisons further substantiate these observations. Group A exhibited a significant decline in resiliency between Day 1 and Day 7, indicating an early reduction in mechanical performance. However, subsequent time intervals showed no significant differences, indicating recovery or stabilization of properties. This aligns with previous findings where surface coating agents like monomers initially reduce surface plasticizer loss but may not completely prevent long-term degradation [12]. In contrast, Group B demonstrated a significantly increasing trend in resiliency from Day 1 through Day 14 and maintained relatively higher values throughout the study period. This suggests that the Soft Liner Kit by Maarc may possess a more robust formulation or better interaction with the monopoly coating, allowing for prolonged effectiveness. The sharp improvement in the first week is particularly noteworthy and clinically advantageous, as this is the period when patients are most sensitive and adaptable [13]. The study by Gardner [14 revealed that Orthoplast Acryton exhibited superior initial resiliency, while the Maarc Soft Liner showed significantly improved and sustained performance from Day 7 onward. Both materials demonstrated comparable resiliency by Day 14, indicating stabilization over time. These findings suggest that monopoly coating effectively enhances short- to medium-term performance of tissue conditioners. The role of a monopoly as a surface coating agent appears promising in both groups, especially in mitigating the common drawbacks of soft liners, such as roughness, stiffness and plasticizer leaching. Although differences between the materials existed in the initial and mid-term phases, by Day 28, both materials demonstrated comparable resiliency. This long-term consistency implies that either brand, when coated with a mono-poly, may offer acceptable performance in clinical use for up to four weeks. These findings reinforce the utility of monopoly coating in extending the functional life and performance of tissue conditioners, particularly when frequent replacements are not feasible. Given that tissue conditioners are often used in post-surgical, interim prosthetic or implant-related phases, maintaining softness and elasticity without compromising structural integrity is critical. This study was conducted under *in vitro* conditions, which may not completely replicate the dynamic oral environment, including temperature changes, microbial activity and masticatory stresses. Additionally, only two commercially available materials were tested. Future *in vivo* studies with larger sample sizes and additional coating materials may offer more comprehensive insights into their long-term clinical efficacy.

## Conclusion:

While Orthoplast Acryton initially exhibited higher resiliency, the Soft Liner Kit by Maarc demonstrated better sustained performance over the 28 days. Both materials, when coated with monomer, showed acceptable long-term resiliency, supporting their clinical applicability for short- to medium-term tissue conditioning.
